# An Unusual Case of Paroxysmal Third-Degree Atrioventricular Block: Thinking Outside the Box

**DOI:** 10.7759/cureus.62782

**Published:** 2024-06-20

**Authors:** Vanessa Sabella-Jiménez, Priscilla Duran Luciano, Carlos Otero-Herrera, Carlos Espiche, Abel J Triana, Sulejman Celaj

**Affiliations:** 1 Department of Medicine, Universidad del Norte, Barranquilla, COL; 2 Department of Public Health, Universidad del Norte, Barranquilla, COL; 3 Department of Cardiology, Albert Einstein College of Medicine, Bronx, USA; 4 Department of Internal Medicine, St. Barnabas Hospital Health System, Bronx, USA; 5 Department of Internal Medicine, Jackson Memorial Hospital, Miami, USA; 6 Department of Cardiology, St. Barnabas Hospital Health System, Bronx, USA

**Keywords:** third-degree av block, paroxysmal av block, adrenergic beta antagonists, acute urinary retention (aur), ophthalmic side effects

## Abstract

During the assessment of a third-degree atrioventricular (AV) block in a patient with syncope, different etiologies should be considered and evaluated. Extrinsic vagal paroxysmal AV block, extrinsic idiopathic AV block and intrinsic paroxysmal AV block are among the types of third-degree AV block in the differential diagnoses. Extrinsic vagal paroxysmal third-degree atrioventricular block (EV-AVB) is linked to parasympathetic influence on cardiac conduction and can be observed in bladder distention and urinary retention. Topical and ophthalmic beta-blockers have shown systemic effects such as bradycardia with and without syncope. We present the case of an 80-year-old male with symptomatic EV-AVB likely precipitated by bladder outlet obstruction and chronic use of an ophthalmic beta-blocker, often overlooked causes of third-degree AV block.

## Introduction

The etiology of a third-degree paroxysmal atrioventricular (AV) block can pose a diagnostic challenge, motivating physicians and specialists to conduct thorough investigations for an accurate diagnosis. Following a patient’s sudden loss of consciousness and other acute syncope-related symptoms, it is crucial for the healthcare professional to classify the etiology by carefully reviewing possible causes. Three types of paroxysmal AV block include intrinsic paroxysmal AV block (I-AVB), extrinsic vagal paroxysmal AV block (EV-AVB), and extrinsic idiopathic paroxysmal AV block (EI-AVB), each presenting different clinical and electrocardiographic features [1].

I-AVB usually manifests in patients with underlying heart disease and is due to an intrinsic disease of the AV conduction system, often referred to as a Stokes-Adams attack or “cardiac syncope” [1]. In contrast, EV-AVB is influenced by parasympathetic activity on cardiac conduction, which plays a role in “reflex syncope” [1]. EI-AVB is associated with low values of endogenous adenosine and is believed to be one of the mechanisms involved in “low adenosine syncope” [1]. However, the presence of one mechanism of paroxysmal AV block does not rule out others [2], posing a challenge in diagnosing certain patients with complex cases.

Topical timolol, a non-selective beta-blocker commonly used for the treatment of open-angle glaucoma, has been reported to produce systemic effects such as bradycardia, third-degree AV block [3], and worsening congestive heart failure [4-6]. The systemic bioavailability of ophthalmic timolol is around 78% compared with oral timolol, which is around 61% due to first-pass metabolism [7]. After administering the ophthalmic solution, the drug can be detectable in plasma after 15 minutes [7]. One drop of 0.5% ophthalmic timolol is estimated to be around 10 mg oral dose [4,8].

We present the case of an 80-year-old male with symptomatic EV-AVB as the primary diagnosis, likely precipitated by bladder outlet obstruction, as well as the synergistic effect of chronic use of timolol, an ophthalmic beta-blocker, representing often overlooked causes of third-degree AV block. Given the challenge of diagnosing paroxysmal third-degree AV block, it is fundamental to consider all contributing factors and thoroughly evaluate the patient’s medical history, symptoms, and test results to determine the most appropriate treatment plan.

## Case presentation

An 80-year-old Caucasian male, with a past medical history of untreated hypertension, dyspepsia, and open-angle glaucoma treated with topical timolol maleate 0.5%, presented to the emergency department with complaints of new-onset lightheadedness, weakness, and loss of consciousness. Approximately six hours prior to his arrival at the emergency department, the patient woke up at night experiencing urinary urgency, accompanied by lightheadedness and weakness. Following voiding, he experienced non-bloody and non-bilious vomiting, abdominal discomfort, and watery, non-bloody diarrhea. He had two brief episodes of unconsciousness, without urinary or fecal incontinence, followed by spontaneous regaining of consciousness after each episode. He denied experiencing palpitations, chest pain, abnormal body movements, tongue bites, or any other symptoms before, during, or after the current episode. He reported no history of smoking, alcohol intake, or illicit drug use.

On examination, the patient was alert, oriented to person, place, and time, and had no neurological deficits. His heart rate was 58 bpm, respiratory rate was 20 rpm, blood pressure was 190/92 mmHg, and saturation of peripheral oxygen (SpO2) was 100% on room air. Rhonchi were noted on auscultation of the right middle lung, and a palpable mass in the hypogastrium was found without rebound or guarding. His cardiovascular exam was unremarkable, with normal S1 and S2 heart sounds and no murmurs, gallops, or rubs. However, he presented decreased heart rate and rhythm.

**Figure 1:**
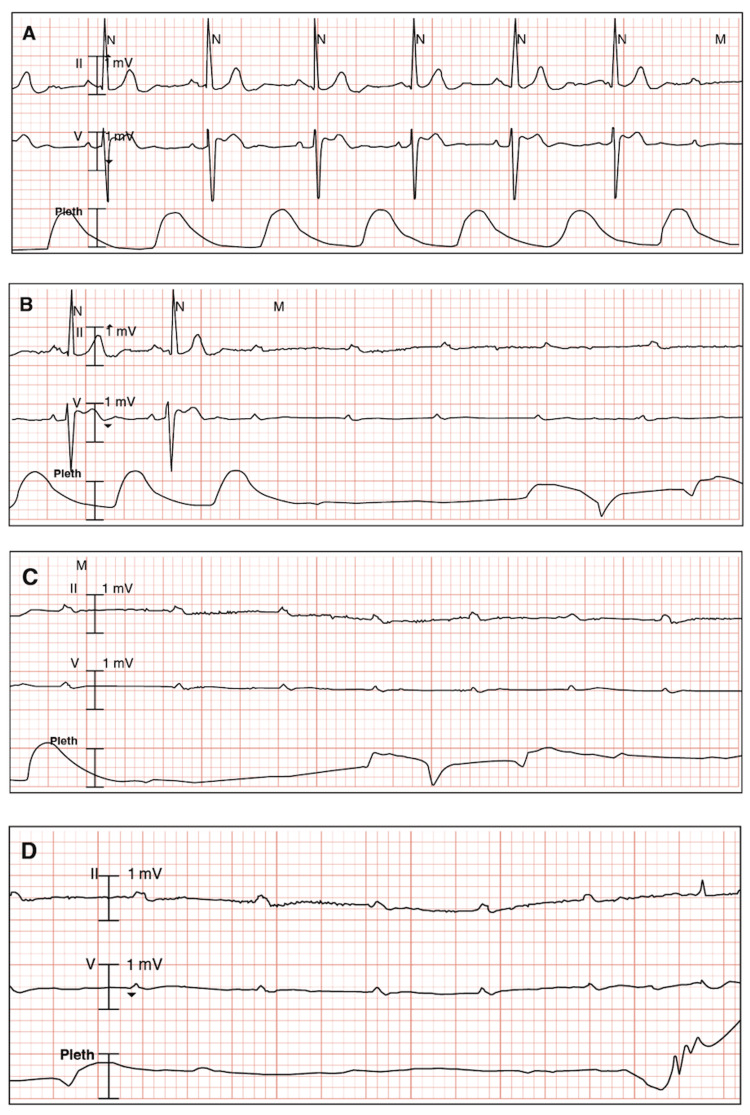
Electrocardiogram findings on continuous monitoring. A. Bradycardia and new-onset third-degree atrioventricular block. B-C. Recurrent episode of third-degree atrioventricular block with ventricular asystole. D. Resumption of third-degree atrioventricular block and ventricular asystole with a narrow QRS complex initiation.

During continuous electrocardiogram (ECG) monitoring, he re-experienced lightheadedness and weakness without losing consciousness. The ECG showed bradycardia with a third-degree AV block, as shown in Figure 1.

His initial laboratory workup showed mild leukocytosis, neutrophilia, hyperglycemia, and elevated levels of prostate-specific antigen and N-terminal pro-brain natriuretic peptide (NT-proBNP), as detailed in Table 1. The remaining electrolyte, hepatic, lipid, and endocrine panels, as well as renal function, were within reference limits.

**Table 1:** Initial laboratory results. NT-proBNP: N-terminal pro-brain natriuretic peptide; TSH: thyroid stimulating hormone; HDL: High-density lipoprotein; LDL: Low-density lipoprotein

Laboratory	Measured Value	Reference Values
Hematologic		
Leukocyte count	12,8000/mm^3^	4500-11,0000/mm^3^
Neutrophils, segmented	88.9%	54-62%
Lymphocytes	5.3%	25-33%
Hemoglobin, blood	14.1 g/dL	13.5-17.5 g/dL (male)
Hematocrit	45.7%	41-53% (male)
Mean corpuscular volume	94.6 μm^3^	80-100 μm^3^
Mean corpuscular hemoglobin	29.2 pg/cell	25-35 pg/cell
Mean corpuscular hemoglobin concentration	30.9% Hb/cell	31-36% Hb/cell
Platelet count	264,000/mm^3^	150,000-400,000/mm^3^
General Chemistry		
Glucose (random, non-fasting)	167 mg/dL	<140 mg/dL
Sodium	139 mEq/L	136-146 mEq/L
Potassium	3.9 mEq/L	3.5-5.0 mEq/L
Chloride	101 mEq/L	95-105 mEq/L
Calcium	10 mg/dL	8.4-10.2 mg/dL
Magnesium	1.7 mg/dL	1.5-2.0 mg/dL
Phosphorus, inorganic	3 mg/dL	3.0-4.5 mg/dL
Urea nitrogen	14 mg/dL	7-18 mg/dL
Creatinine	0.58 mg/dL	0.6-1.2 mg/dL
Hepatic		
Proteins, total	7.3 g/dL	6.0-7.8 g/dL
Albumin	4.4 g/dL	3.5-5.5 g/dL
Bilirrubin, total	0.6 mg/dL	0.1-1.0 mg/dL
Aspartate aminotransferase	27 U/L	12-38 U/L
Alanine aminotransferase	31 U/L	10-40 U/L
Alkaline phosphatase	90 IU/L	25-100 U/L
Lipase	308 U/L	0-160 U/L
Lipids		
Cholesterol, total	176 mg/dL	< 200 mg/dL
HDL	50 mg/dL	40-60 mg/dL
LDL	112.4 mg/dL	<160 mg/dL
Triglycerides	68 mg/dL	< 150 mg/dL
Endocrine		
TSH	2.38 μU/mL	0.4-4.0 μU/mL
Free T4	1.22 ng/dL	0.9-1.7 ng/dL
Others		
Lactic acid	1.9 mmol/L	0.5-1 mmol/L
Prostate-specific antigen	13.2 ng/ml	1.0-1.5 ng/ml
Creatine phosphokinase	40 U/L	39 – 308 U/L
NT-proBNP	200 pg/mL	<125 pg/mL
Troponin I	<0.012 ng/ml	<0.04 ng/mL
Urinalysis	Normal	Normal
Toxicology, urine	Negative	Negative

**Figure 2:**
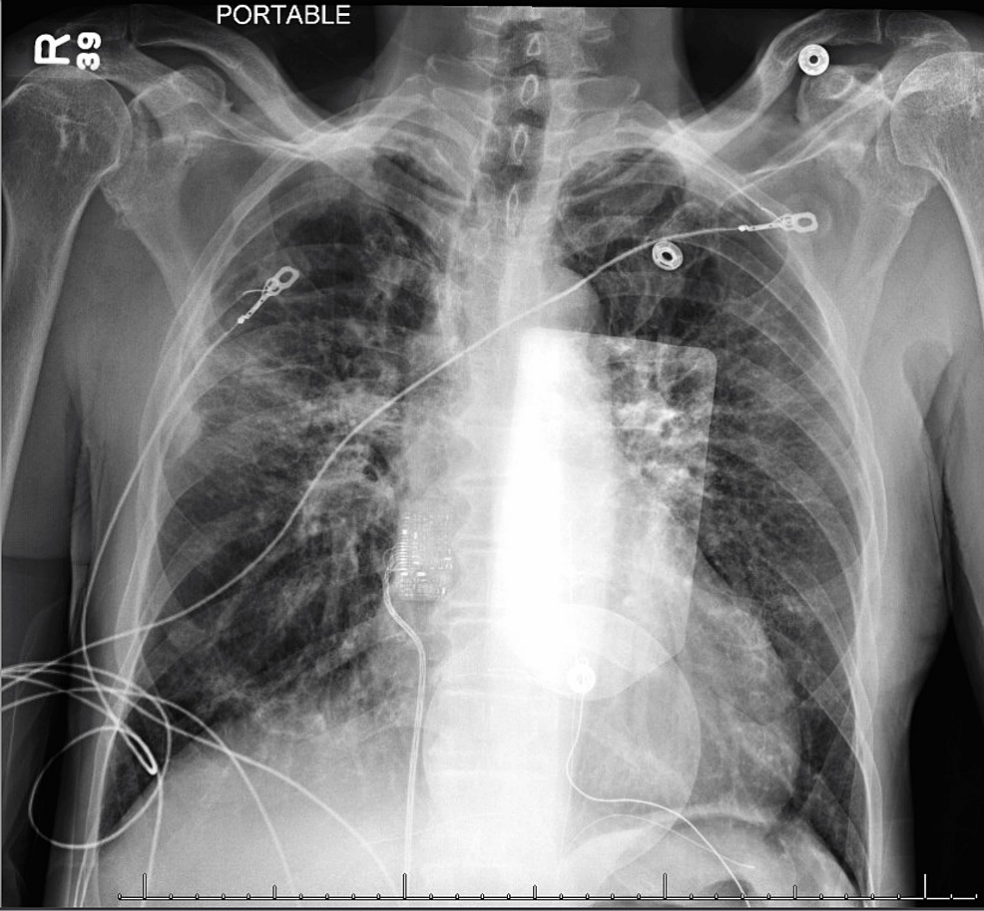
Chest X-ray with bilateral perihilar interstitial indistinctness and focal opacity in the right middle lung.

The initial chest X-ray revealed bilateral perihilar interstitial indistinctness and a focal opacity in the right middle lung, indicative of edema vs infectious process (Figure 2).

A head CT scan showed no signs of acute intracranial hemorrhage, midline shift, or mass effect. However, it revealed images consistent with chronic small vessel ischemic changes and old infarcts. The transthoracic echocardiogram showed normal-sized four chambers with no septal defects or pericardial effusion. Left ventricular systolic function was normal, with an ejection fraction of 60-65%. Right ventricular systolic function and pressure were also normal. The septal motion was consistent with a conduction abnormality, and there was evidence of mild aortic sclerosis.

**Figure 3:**
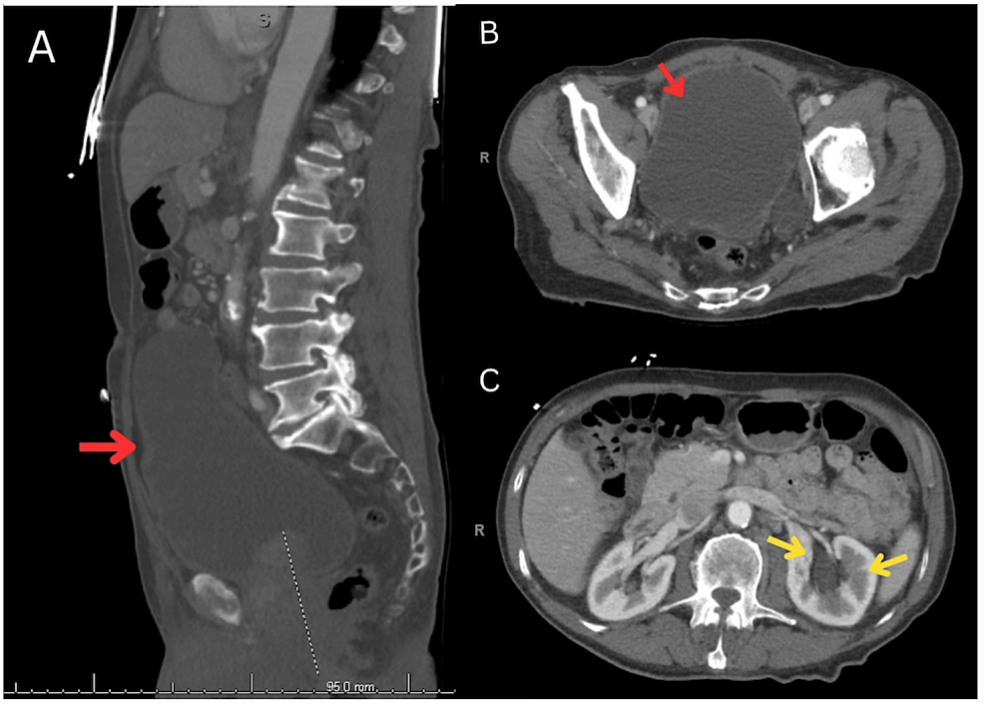
Abdominal CT scan. A. Sagittal view showing marked bladder distention (red arrow). B. Axial plane with markedly distended bladder (red arrow). C. Axial plane with moderate left hydroureteronephrosis (yellow arrows) and mild right hydronephrosis.

The abdominal ultrasound evidenced moderate left and mild right hydronephrosis, trace ascites, central prostatic gland hypertrophy, and bladder outlet obstruction. A CT scan of the abdomen and pelvis showed a noticeably distended bladder with chronic outlet obstruction sequelae, including diverticula and wall trabeculations, secondary to marked prostatomegaly, as seen in Figure 3A-3B. Additionally, the CT scan showed moderate left hydroureteronephrosis (Figure 3C) and mild right hydronephrosis secondary to the bladder outlet obstruction.

A Foley catheter was inserted, draining more than 2000 ml, which relieved the bladder outlet obstruction and contributed to clinical improvement. As part of the medical management, topical timolol was discontinued and latanoprost 0.005% ophthalmic solution was initiated. The patient was started on lisinopril 10 mg orally daily, due to elevated systolic blood pressure and the need to avoid other nodal blocking agents. Serial abdominal ultrasound and chest X-ray imaging were unremarkable confirming clinical improvement. However, the patient stayed in the coronary care unit for monitoring while awaiting placement of a permanent pacemaker due to his recurrent episodes of symptomatic third-degree AV block.

A Medtronic (Medtronic plc, Dublin, Ireland) leadless VDD pacemaker (ventricle paced, atrium and ventricle sensed, dual operation) was placed with no complications, and with stable, underlying normal sinus rhythm and sensing capture thresholds, with no subsequent episodes. Urology initiated tamsulosin and finasteride and continued his outpatient workup. At his follow-up visit with electrophysiology, the patient reported no symptoms. His pacemaker was interrogated, with no changes performed to his device settings (VDD 50-130). No ventricular episodes were reported. Stable sensing and capture thresholds were present.

## Discussion

The present report illustrates how unusual cumulative risk factors can precipitate an electrical conduction disorder. Based on the patient’s age, urinary presentation, and laboratory and ECG findings, EV-AVB was considered the primary diagnosis, while the influence of the ophthalmic beta-blocker was seen as a potential contributor of systemic and secondary effects, prompting its discontinuation. 

EV-AVB occurs when a vagal input depresses sinus node function and AV nodal conduction without influencing the velocity of conduction in the His-Purkinje system, causing the site of the EV-AVB to be within the AV node [9]. EV-AVB is linked to parasympathetic influence on cardiac conduction [1]. In the present case, it is possible that stimulation of mechanoreceptors at the base of the bladder wall, as a result of elevated intravesical pressure, transferred signals to the vagal nuclei via the hypogastric and sacral plexus nerve [10]. This process likely increases parasympathetic tone [10] and causes a sudden modification in vascular resistance and/or heart rate [11].

Furthermore, chronic use of an ophthalmic beta-blocker may have exacerbated the bradycardic effects of this vagal stimulation, leading to the symptomatic bradycardia experienced by the patient. Vagal nerve stimulation from pelvic organs has been primarily reported in patients with substantial bladder distention and urinary retention, causing symptomatic third-degree AV block [11] and syncope due to ventricular tachycardia [12]. EV-AVB is associated with autonomic activation symptoms such as lightheadedness and nausea [1], as well as vagal overactivity symptoms such as vomiting, which often occur during the night [9], similar to the symptoms experienced by the patient.

The diagnosis of EV-AVB is made in the presence of a simultaneous progressive slowing of the sinus rate (P-P cycle increases) with PR interval prolongation before initiation of complete AV block, followed by ventricular asystole and sinus rate slowing (P-P cycle increases), and finally resumption of AV conduction on sinus acceleration (P-P cycle decreases) [9]. In these cases, there is an absence of structural heart disease [1], as confirmed by the transthoracic echocardiogram of this patient. Likewise, prodromes are always present and last longer than 10 seconds [1], which were experienced by the patient prior to several syncopal episodes. Additionally, while the history of syncope is long and the onset can occur at any age [1], in the present case the syncope onset was acute in an elderly patient.

Ophthalmic timolol, a non-selective beta-adrenergic antagonist, has been known to cause AV block, even after 13 years of consistent and chronic use [3]. In this case, the patient confirmed the use of ophthalmic timolol for more than five years. Initially, topical timolol reaches the nasal mucosa and is eventually absorbed at 80% into the systemic circulation, bypassing the first-pass effect [13] and contributing to conduction block and syncope [14,15]. Our case highlights the importance for clinicians to consider the possible side effects of medications, regardless of the route of administration, as well as all individual patient risks when evaluating a patient with an electrical conduction alteration. Individuals taking ophthalmic timolol should be more closely monitored [16] since the cardiovascular side effects are often overlooked yet can be life-threatening [3]. Additionally, in elderly patients, the beta-receptor antagonist effect of ocular timolol after a single dose is strong and long-lasting, and the elimination is much slower than in a healthy young adult, which explains the reported systemic side effects of ophthalmic timolol [17]. In this case, a prostaglandin analog should be considered as the first-line treatment [4], with the discontinuation of an ophthalmic beta-blocker being advised. 

I-AVB and EI-AVB were among the excluded conduction disease etiologies. I-AVB was ruled out since the patient had no intrinsic disease of the AV conduction system, underlying heart disease, bundle branch block, or wide QRS complexes [1,18]. I-AVB usually initiates with an atrial or ventricular premature extrasystole, sinus slowing, and unchanged PR interval, is followed by ventricular asystole with an increase in sinus rate, and is terminated by a premature ventricular beat with resumption of AV conduction [9], which were not observed in the ECG during continuous monitoring. Additionally, in I-AVB prodromes are typically absent or very short duration (< 5 seconds) [1], which does not correlate with the symptoms presented.

Excluding EI-AVB was a challenge since the co-existence of two types of mechanisms, i.e. vagal and adenosine, may be observed [1]. EI-AVB, like EV-AVB, is characterized by normal AV conduction, absence of structural heart disease, and narrow QRS complexes [1]. While EV-AVB is typically associated with well-identifiable triggers and characteristic symptoms of autonomic activation (lightheadedness, dizziness, nausea) [19], EI-AVB is characterized by low values of endogenous adenosine. Furthermore, in EI-AVB there is an unchanged sinus rate (before, during, and after the AV block) [1,9], which is similar to the ECG findings of the patient. However, due to the resolution of symptoms after the bladder outlet obstruction was addressed and the ophthalmic timolol was discontinued, EV-AVB was considered the primary diagnosis.

Finally, while pacemaker therapy is partially effective in EV-AVB since syncope can recur, cardiac pacing is the most effective therapy in this scenario [1]. For patients with symptomatic bradycardia presumed to be due to an acquired third-degree AV block, permanent pacing is a Class I recommendation (Level of evidence C) [20]. In this specific case, the pacemaker had been effective for four years with no recorded recurrence.

## Conclusions

The combination of new-onset bladder outlet obstruction and chronic ophthalmic beta-blocker use can contribute to the development of EV-AVB. However, these factors are often overlooked and rarely considered as causative agents for third-degree AV block. In conclusion, the presented case highlights the importance of recognizing and addressing uncommon yet significant interactions between medication use, bladder function, and cardiac conduction, which emphasizes the necessity for a comprehensive and interdisciplinary approach in managing complex clinical scenarios.
